# Supplementary material for: Insomnia, Daytime Sleepiness, and Quality of Life among 20,139 College Students in 60 Countries around the World—A 2016–2021 Study
Source: J Clin Med. 2023 Jan 15;12(2):692. doi: 10.3390/jcm12020692 (PMC9866097; doi:10.3390/jcm12020692)
Supplement: Supplementary file 1 [file jcm-12-00692-s001.zip › jcm-2144818-supplementary.pdf]

Table S1. Comparison of the mean scores of the individual MANSA questions according to insomnia and excessive daytime sleepiness.

Question	AIS				ESS			
	Insomnia	No insomnia			Daytime sleepiness	Normal		
	M SD	M SD	Cohen's d	p	M SD	M SD	Cohen's d	p
How satisfied are you with your life as a whole today?	4.23 ± 1.36	5.00 ± 1.8	3.84	<0.001	4.31 ± 1.38	4.65 ± 1.32	0.25	<0.001
How satisfied are you with your job (or sheltered employment, or training/education as your main occupation)?	4.18 ± 1.44	4.76 ± 1.26	0.43	<0.001	4.21 ± 1.46	4.51 ± 1.37	0.22	<0.001
How satisfied are you with your financial situation?	3.82 ± 1.60	4.39 ± 1.46	0.35	<0.001	3.80 ± 1.60	4.15 ± 1.55	0.22	<0.001
How satisfied are you with the number and quality of your friendships?	4.54 ± 1.75	4.93 ± 1.63	0.23	<0.001	4.56 ± 1.75	4.76 ± 1.69	0.12	<0.001
How satisfied are you with your leisure activities (hobby)?	3.91 ± 1.66	4.54 ± 1.54	0.39	<0.001	3.91 ± 1.68	4.27 ± 1.62	0.22	<0.001
How satisfied are you with your accommodation?	4.60 ± 1.66	5.03 ± 1.49	0.27	<0.001	4.60 ± 1.66	4.85 ± 1.57	0.15	<0.001
How satisfied are you with your personal safety?	4.98 ± 1.49	5.53 ± 1.27	0.40	<0.001	4.99 ± 1.49	5.30 ± 1.39	0.21	<0.001
How satisfied are you with the people that you live with?	4.88 ± 1.63	5.28 ± 1.49	0.26	<0.001	4.86 ± 1.64	5.12 ± 1.53	0.16	<0.001
How satisfied are you with your sexual life?	3.96 ± 1.96	4.46 ± 1.89	0.26	<0.001	4.02 ± 1.98	4.22 ± 1.93	0.10	<0.001
How satisfied are you with your relationship with your family?	4.77 ± 1.55	5.26 ± 1.33	0.34	<0.001	4.80 ± 1.57	5.05 ± 1.44	0.17	<0.001
How satisfied are you with your physical health?	4.02 ± 1.51	4.79 ± 1.36	0.54	<0.001	4.02 ± 1.54	4.46 ± 1.46	0.29	<0.001
How satisfied are you with your mental health?	3.69 ± 1.67	4.80 ± 1.52	0.70	<0.001	3.74 ± 1.68	4.32 ± 1.67	0.35	<0.001

M – mean; SD – standard deviation; AIS – Athens Insomnia Scale; ESS – Epworth Sleepiness Scale;
